# A smart medicine reminder kit with mobile phone calls and some health monitoring features for senior citizens

**DOI:** 10.1016/j.heliyon.2024.e26308

**Published:** 2024-02-15

**Authors:** Liton Chandra Paul, Sayed Shifat Ahmed, Tithi Rani, Md Ashraful Haque, Tushar Kanti Roy, Md Najmul Hossain, Md Azad Hossain

**Affiliations:** a Dept. of Electrical, Electronic and Communication Engineering, Pabna University of Science and Technology, Pabna-6600, Bangladesh; b Dept. of Electronics and Telecommunication Engineering, Rajshahi University of Engineering and Technology, Rajshahi-6204, Bangladesh; c Dept. of Electrical and Electronic Engineering, Daffodil International University, Dhaka, Bangladesh; d Dept. of Electronics and Telecommunication Engineering, Chittagong University of Engineering and Technology, Chittagong, Bangladesh

**Keywords:** Smart medicine reminder box, Medication safety, Low cost, Health monitoring, Microcontroller, Real-time scheduling, GSM module

## Abstract

The demand for an effective system that combines cutting-edge technologies with medical research to improve healthcare systems has increased with the development of medical technology. The most fundamental form of disease prevention is taking the right medication when needed. With the right care, many fatal diseases can be cured or prevented. Therefore, it is crucial to follow the doctor's recommended drug plan. Healthcare experts now have serious concerns about patients not being able to take their prescribed medications on time, particularly elderly patients. Due to age-related memory loss, people who have been given multiple prescriptions at once over an extended period of time are more likely to forget to take their medication on time or to take the wrong medication. Sometimes, a patient's inability to take the right medication at the right time might have a major impact on their health. Aside from being forgetful, patients, especially the elderly and illiterate, may not be able to read the name stated on medical containers, leading to the consumption of the wrong medication. These errors contribute to non-adherence to pharmaceuticals, which is detrimental to the patient's health. As a result, there is a significant problem that hinders the success of the treatment. The medication reminder system is intended for people who frequently take medications or vitamin supplements in order to handle this. In order to help an elderly person properly take their medication and help the patient have a healthy life, we have created a ground-breaking portable multifunctional medicine reminder kit with phone calls. Other intelligent characteristics of the smart medicine reminder include the capacity to show the time, date, and day in real time, the detection of smoke, the measurement of air humidity and temperature in the room, the measurement of heartbeats per second, the patient's body temperature, and the oxygen saturation level.

## Highlights

• Not only acts as a smart medicine reminder kit but also provides some health monitoring features.
• Completely eliminates the potential for medication errors.
• Very low manufacturing cost, portable, and user-friendly operation.
• Though it is mainly designed and tested in home settings, it can also be extended to support doctors and nurses in hospital settings.
• During a one-month practical trial, it performed flawlessly and extremely well.

## 1 Introduction

Due to the prevalence of many diseases among the people of Bangladesh, a huge number of them are forced to take medicine regularly. The affection rate also increases daily. Though some diseases may not cause severe suffering, some are fatal. Some diseases can be controlled by taking medicine regularly and on time. The lifespan of human beings is affected by those diseases. We need to take many medicines regularly for survival [1]. In most circumstances, people's memories start to decline as they age. Numerous people require ongoing assistance, whether they are our elderly or particularly abled people. The timing of taking a prescribed dose has a greater influence on elderly patients than on others; for this reason, taking the right medication at the right time is crucial to preventing future complications and curing the illness. According to studies, between 40% and 75% of adults fail to take their prescribed medications on time each day. As a result, individuals fail to take the right dosage at the right time, occasionally administer the incorrect dosage, or utilize outdated medication, which may result in additional patient suffering and occasionally put their lives in danger [2].

In order to eliminate the aforementioned risks as well as mitigate the requirement of continuous observation to take the scheduled dose, we are motivated to search for an easy, user-friendly, and effective approach. Some types of medicine kits have already been proposed by some researchers, but most of them are not user-friendly for senior citizens. Some of them are not portable due to their larger size. Nowadays, human errors in many sensitive sectors have been reduced by using modern programmable wireless technologies. The use of modern automotive technologies enhances accuracy, so if we merge the concept of programmable modern wireless technology with the medicine reminder kit, then it will be able to provide great features, including reducing size. Different types of medical systems have been previously proposed upon different platforms and concepts, for example: an automated reminder medicine box [3], med-assist [4], medication reminders [5,6], a hybrid automatic reminder [7], and a medication assistive system [8]. One of the main obstacles to the widespread adoption of e-health care technology in practice is that some of the systems lack user-friendly interfaces for both medical professionals and patients. Some systems are wholly app-dependent, which just serves to remind users by delivering brief messages, while others are totally database-related, work using RF technology, and have Bluetooth connectivity. But it's fairly usual to merely alert the user when they need to take medication; we need something simple, new, and more intelligent, with more functions. There are essentially two types of medication reminders in the literature: software-based and hardware-based. The majority of software-based methods are not appropriate for elderly and illiterate rural residents [4]. A. Mathew et al. [9] implemented a medicine dispenser with a good touch system to input doctors prescribed schedules for taking medication. The system does not have a phone call alert function or other health monitoring features. A medicine reminder system has also been discussed in Ref. [10]. The system is capable of creating audio-visual alerts, including an email sent to the patient's email address. Our proposed medicine reminder system makes a bridge between hardware and software, which delivers it as a smart and user-friendly system with some attractive features. In Ref. [11], a pill box reminder system has been designed using a real-time clock interface and microcontroller. The system reminds the patient by playing only the alarm at the scheduled time. On the other hand, we have integrated a GSM system module with our proposed smart system, which is able to make a phone call after a few minutes if the patient does not open the door of the medicine box after playing an alarm. We have also used an ESP32 module to keep track of when a patient should take medicine. LED lights are used as indicators to open medicine boxes. In Ref. [12], an intelligent system built on the principles of reinforcement learning and deep learning is presented that automatically aids patients who must adhere to a prescribed treatment regimen at home. When it is necessary to consume a dose of medication, the system may aid the individual by sending personalized reminders as well as warnings to help prevent medication mistakes in the event that the patient accidently takes the wrong drug. The system, which consists of three major agents, can self-tune depending on the patient's abilities. Due to some inaccurate categorizations, it displays a flawed identification. It calls for the system to be retrained in regard to new medications not taken into consideration as well as pre-trained in regard to pillbox images. Another assistance system [13] based on home sensors, ambient intelligence, and artificial intelligence is proposed to overcome the shortcomings of [12] as well as improve the functionality of both the tutor and checker. To reduce medication errors, this system will remind the patient about their treatment plan and make sure they take the proper prescription at the right time. While our proposed system incorporates capabilities for monitoring the patient's heart rate, body temperature, and oxygen saturation level, smoke detection, poisonous gas detection, room temperature estimation, and humidity estimation, both works illustrated in Refs. [12,13] lacked all of those health monitoring features. One prototype's implementation costs are quite minimal (approximately 32 USD). It will be greatly decreased if the suggested system is produced commercially for sale. As a result, our suggested solution offers a smart medicine reminder system and some unique health monitoring features, together with very consistent performance at a very low cost.

Our proposed smart medicine reminder kit is specially designed for senior citizens. Initially, the system needs to store medicines as per the doctor's suggestion in all boxes of the kit and receive input for the medicine routine through any mobile phone. The reminder system can sound an alarm at the scheduled time, which can be set by mobile phone as per a doctor's prescription. If the patient does not take medicine after being alerted, the smart kit will make repeated phone calls to the patient's number until the medication is taken. The proposed smart system also eliminates the chance of taking the wrong or expired medicine. Some medicine boxes had a feature to send messages instead of phone calls. But sending messages is not convenient because there are many users who ignore the messages on their mobile phones. Moreover, sending a message to inform the user is a very short notification compared to a phone call. So users only need to save the SIM number used in the medicine reminder kit on their cell phone so that the patient can easily understand why he has been given the phone call. Using the proposed smart system, senior citizens can easily avoid missing their daily medication schedule. Though self-medication is the best solution, sometimes it is difficult for a senior citizen due to many aging difficulties. Accordingly, we have developed a smart medicine reminder kit. It also includes some other features, like heart rate measurement and an oxygen monitoring system that can be easily seen on an LCD display, resulting in the patient being informed of his or her present condition. In this COVID-19 situation, it is the most important feature so that the patient or other family member can easily take proper steps before the condition turns serious. Furthermore, room temperature and air quality can also be monitored by the ATmega328P Arduino microcontroller. Whenever the air quality drops below the threshold value, an audible message informs the user about the environment's conditions. So basically, it is not only a traditional medicine reminder box; its additional useful features make it a smart medicine reminder kit. This home medical interface is necessary to assist in self-medication management and warn the elderly recipient about incorrect medication.

Our primary contribution is the creation of a senior citizen-friendly, low-cost smart drug reminder kit with calls from mobile phones and some extra health monitoring capabilities. Additionally, we created an app that allows users to enter the suggested kit's medication schedule. Medication errors will happen only when a doctor prescribes the wrong medicine; otherwise, our suggested technique completely eliminates the potential for drug errors. Because there is no possibility of opening the incorrect medicine chamber, only one proper medicine chamber is unlocked at a time as per the input of the doctor's prescription. Practical tests revealed that our suggested and created kit performed flawlessly and extremely well. Our proposed kit has mainly been designed and tested in home settings. However, it can extend to supporting doctors and nurses in hospital settings. When an emergency patient needs to stay at the hospital for some days and needs to be fed medicine frequently and in a timely manner, this proposed system can be deployed for the patient, and at every prescribed time, the assigned doctor or nurse will get an alarm, i.e., a reminder, which reduces the stress of keeping track of a large number of patients' schedules.

## 2 Conceptualization and system modeling

**Fig. 1:**
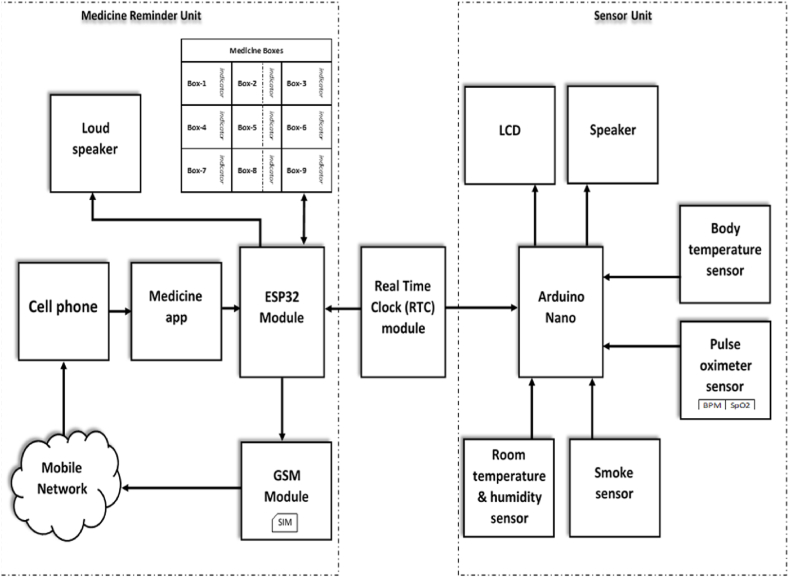
Block diagram of the proposed smart medicine reminder kit for senior citizens.

The World Health Organization (WHO) estimates that more than 80% of adults (aged 50 to 60) take medication two to three times per day [14]. Among them, 40–60% of individuals forget to take prescribed medication at the appropriate time. Many old people and even some young people suffer from common disorders, including low or high blood pressure, diabetes, gastritis, hypocalcemia, etc., which necessitate the frequent use of common medications in order to maintain a normal quality of life [15]. Patients can forget to take the proper medication at the right time or may take the wrong medication due to the aging effect, which can result in serious health risks or prolong the patient's recovery period. This happens more frequently in older patients. We can fix all the aforementioned problems by creating an intelligent, programmable electronic medicine reminder kit. The block schematic in Fig. 1 represents the idea behind our smart drug reminder kit. Not only does this suggested kit remind patients to take their medications, but it also facilitates other services like making repeated phone calls to the patient if they fail to do so after receiving an alarm, measuring the temperature and humidity of the room, taking the patient's temperature, detecting smoke, and measuring their heart rate and oxygen saturation level.

The sensor unit and the medication reminder unit are the two main components of the proposed electronic system. An airtight box with nine airtight compartments serves as the structure of the medicine reminder unit. Each chamber has an LED installed inside it to serve as an indicator as to which chamber has to be opened when the alarm goes off. To display welcome messages and other information, there is an LCD monitor.

The intelligent programmable electronic medicine reminder was created with two goals in mind: to offer a service that is user-friendly, effective, and packed with features, and to lower construction costs. In the suggested system, a nice balance between hardware and software has been established. The ATmega328p microcontroller chip and ESP32 module are used to connect the hardware parts. To input medication schedules in accordance with a doctor's prescription, a specific and well-structured mobile app is created. To input the medication schedule, just link the app to the medication reminder device via any smartphone's WLAN connectivity. To alert the user by playing an alarm at the proper moment, all input planned data is kept in the ESP32 module and is constantly compared to the real-time clock value. If the user forgets to take the medication at the appointed time, the system will immediately contact the patient number they gave in the app via the GSM module, using the kit number from the medication reminder, to remind them to take their medication. It is not entirely dependent on the cellular network, despite the fact that many other projects are. This reminder tool can be used as a primary health monitoring kit in addition to serving as a reminder. It includes a sensor unit with the LM35, MAX30100, DHT11, and MQ-2 sensors, allowing for the measurement of the user's body temperature, BPM, SpO2, air quality, and weather forecast functions. The ATmega328P microcontroller chip is the only one that coordinates and displays all sensor data on the LCD screen.

## 3 Working procedure

**Fig. 2:**
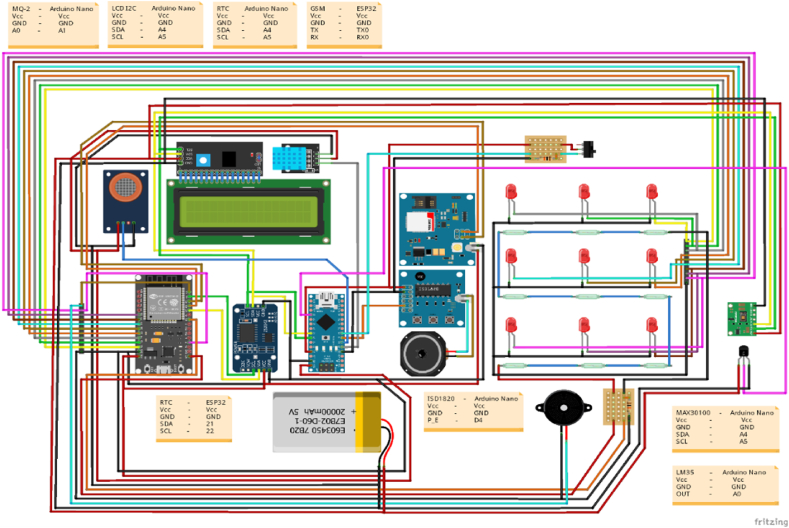
Circuit diagram of smart medicine reminder kit.

**Fig. 3:**
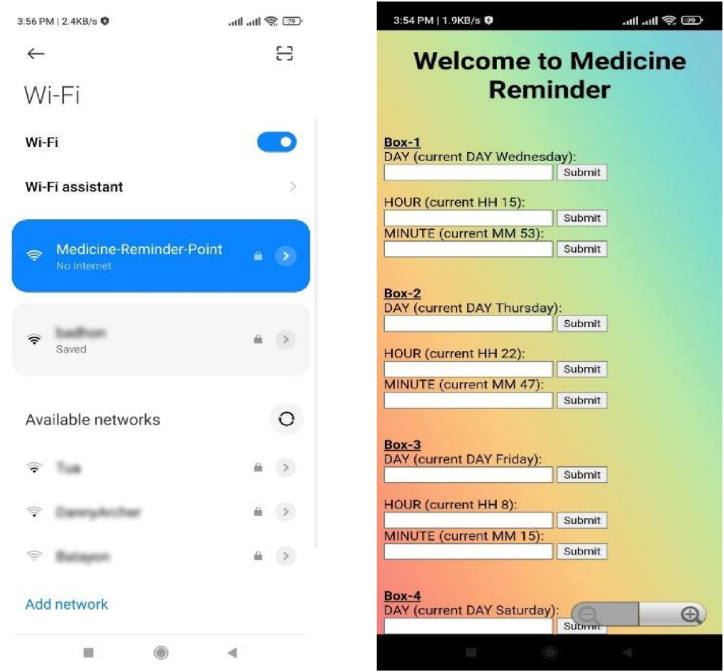
Window of connecting the app to the medicine reminder kit and input the medicine schedule.

Using “Fritzing” software, a schematic circuit diagram of the proposed smart medication reminder system has been created, as seen in Fig. 2. The “Med Alert” smartphone app, which allows the patient or user to input the schedule of taking medication as per a doctor's prescription, controls the smart medicine reminder kit. The app was created using the Java programming language and the “Android Studio” tool. Users must first install the “Med Alert” mobile application before opening settings and entering the WLAN to locate the “Medicine Reminder Point” wireless network, which they must join in order to use the system. After successfully connecting with the medicine reminder kit, the connection will be established after entering an authenticated password in a protected local WiFi access, and it is then available for usage. The Med Alert app's window is depicted in Fig. 3. The medicine reminder unit and the sensor unit are two main components that make up the system's overall framework.

### 3.1 Medicine reminder unit

The medication reminder unit consists of an ESP32 module, a GSM module with a SIM, a loudspeaker, a real-time clock module, several medicine chambers, including a buzzer, an LED indicator, and a reed switch acting as an input signal controlled through a mobile application. The heart of the proposed system is the ESP32. The reminder box has nine airtight chambers to preserve different kinds of medicine for every prescribed time of the day. One LED is present in each chamber, indicating the precise medicine alarm at the designated reminder time. To illuminate the appropriate LED and medicine chamber that is to be eaten at that specific time, the ESP32's GPIO is connected to LEDs. The ESP32's GPIO is also connected to the buzzer. The buzzer acts as an audible cue for the patient to remember to take the specific medications.

A real-time clock (RTC) and GSM900A module with a SIM are attached to the microcontroller ESP32. As counting time is an important issue, the DS3231 RTC module is a savior. It is used for data logging, clock building, time stamping, timers, and alarms. An RTC interface always keeps track of real-time, irrespective of the power supply. To interface with the RTC module, the SDA and SCL pins of the ESP32 microcontroller are connected to the SDA and SCL pins of the RTC, respectively, by the I2C protocol.

The module is operated by a +5V power supply, and a higher voltage may damage it. The RTC module provides information about time and date through serial communication and updates the information within ESP32 in real-time. Usually, the information is exchanged from byte to byte. The libraries that are prepared for the DS3231 module make communication easy. When the header file is incorporated, the controller communicates automatically and provides the date and time. The alarm clock can also be set or changed easily using libraries. If the main power goes out, the RTC module draws power automatically from the coin-cell battery, which will update the time. Therefore, when the whole system restarts, the microcontroller ESP32 gets the real time from the module without error. Refer to the chart below for a quick overview of the connections with the ESP32 microcontroller.

**Table:** Overview of the connections between RTC module and ESP32

RTC Module	ESP32
SDA	SDA (default is GPIO 21)
SCL	SCL (default is GPIO 22)
GND	GND
VCC	3.3V or 5V

The microcontroller ESP32 has a serial peripheral interface flash file system (SPIFFS). SPIFFS is a lightweight file system created for microcontrollers with a flash chip that are connected by the SPI bus, like the ESP32 flash memory. i.e., which is used for storing larger amounts of data in the form of files. By default, about 1.5 MB of the onboard flash is allocated to SPIFFS. The prescribed medicine schedule is stored in a SPIFFS file; every time a new value is sent through a mobile app, the value is stored in a SPIFFS file inside the ESP32 module and displayed in the mobile app interface. The microcontroller ESP32 module stores the medicine information, and the system continuously compares the scheduled time with the actual time by using the RTC module. When the system finds a match between the scheduled time and the current time, the LED of the respective medicine chamber indicating which medication to take is turned on, and a parallel loudspeaker plays the alarm for a certain period [10,16]. The designed medicine system also detects whether scheduled medicine is taken or not. The system generates a phone call reminder if scheduled medicine is not taken by the patient. If the user takes scheduled medicines after the alarm, the action is detected by using a door sensor (reed switch) in each medicine chamber. A voltage-divider circuit is used to establish the interface between the reed switch and the Arduino. The switch is opened for +5 V and closed for 0 V [17]. The response of the reed switch is processed by the microcontroller ESP32. If the scheduled medicine chamber is opened, the LED of the particular medicine chamber is turned off, and it does not send phone calls to the patient number.

If the patient stays in a remote place from the medicine reminder system, then the patient can not see the indicator and cannot hear the alarm. Therefore, the patient may be unable to take the prescribed medicine. In that case, the proposed reminder system waits for 5 min, and then it automatically generates a phone call through the GSM900A module to the patient's number. Here, GSM900A uses the USART protocol to communicate with the ESP32 via TX and RX pins. Its maximum current-carrying capability is 2 A. The RX and TX pins of the GSM module are connected to the RX0 and TX0 pins of the ESP32 microcontroller, respectively.

### 3.2 Sensor unit

Some intriguing characteristics, like measures of heart rate, oxygen saturation level, and body temperature, are provided by this device. Additionally, it has the ability to monitor environmental factors, including smoke, humidity, and room temperature. Sensing devices gather the data, which is then transmitted to the Arduino's processor for processing. The brains of the device are the Arduino Nano. There are three sensors: the MAX30100 sensor, used to measure the patient's blood oxygen level and heart rate [18], the DHT11 sensor, which gauges the environment's temperature and humidity, and the MQ-2 sensor, which detects smoke.

Every time the device is turned on, it first verifies the mode. There are two operating modes for the system: default mode and medical checkup mode. The smart reminder system's default settings include reminder activities, time and date display, smoke detection, room temperature measurement, and humidity measurement. On the other hand, it can measure the heart rate, the amount of oxygen saturation in the blood, and the body temperature when the medical checkup mode is engaged. To switch the mode of operation, use a switch. As indicated, the reminder system keeps functioning internally while in medical checkup mode, serving the principal objective of the proposed device without interfering with the medication reminder system.

#### 3.2.1 Default mode

An RTC module communicates with the Arduino Nano when it is in default mode. On the LCD display, there is a real-time clock and a date with day in the forms HH:MM and DD/MM/YY—day of the week [19]. We can easily use this module on a variety of master devices because RTC communicates via the I2C bus. This indicates that the RTC module is now communicating with the ESP32 and Arduino Nano. The following describes how to connect the RTC module to the Arduino in a fairly simple manner.

**Table:** Overview of the connections between RTC module and Arduino Nano

RTC Module	Arduino Nano
SCL	A5
SDA	A4
VCC	5V
GND	GND

As in this work, we want to display status messages and sensor readings, so it is necessary to interface LCD displays with the microcontroller of the Arduino Nano. Wiring an I2C LCD is much easier than a standard LCD. We just need to connect four pins instead of twelve. As we are using multiple devices on the same I2C bus, we have to set a different I2C address for each of the boards so that it does not conflict with another I2C device. Humidity and room temperature are common parameters for measuring environmental conditions. A sensor called DHT11 has been deployed to measure both humidity and temperature in the environment. The processes of measuring these parameters can be presented as three steps: sensing humidity and temperature in the environment, extracting respective values from the output of the sensor, processing them, and sending them to a 16 × 2 LCD display. In order to detect smoke, a MQ2 gas sensor is installed, and the analog output of the MQ2 gas sensor is again used as the input of a high-precision comparator (LM393) to convert into digital form. Finally, the findings of the gas sensor are also displayed on the LCD display.

#### 3.2.2 Medical checkup mode

In medical checkup mode, the smart medicine reminder kit can assist us in checking three health condition monitoring features of a person: heartbeat rate per minute (BPM), oxygen saturation level (SpO2), and body temperature. Generally, blood pressure monitors are larger in size and require assistance to use. In our prototype, a person just needs to hold the finger on the sensors for a while. In less than 1 min, our prototype is able to inform the user of the heart rate, oxygen saturation level, and body temperature through the LCD display. A pulse oximeter MAX30100 is used in the designed system, which has two lights: one is red and the other is infrared. For oxygen level measurement, both lights are needed. On the other hand, only infrared light is needed for heartbeats per minute (BPM). When the heart pumps the oxygenated blood, the finger is slightly swollen, and less light is detected by the detector. And when the heart relaxes, i.e., receives deoxygenated blood, the detector receives comparatively more light [20]. By calculating the time difference between two states, the heartbeat per minute is determined. The microcontroller processes the measured BPM and oxygen saturation level of the patient and displays the alphanumeric value on the LCD display. The MAX30100 module communicates with the Arduino Nano by following the wiring setup.

**Table:** Overview of the connections between MAX30100 module and Arduino Nano

MAX30100 Module	Arduino Nano
Vin	5V
GND	GND
SCL	A5
SDA	A4

**Fig. 4:**
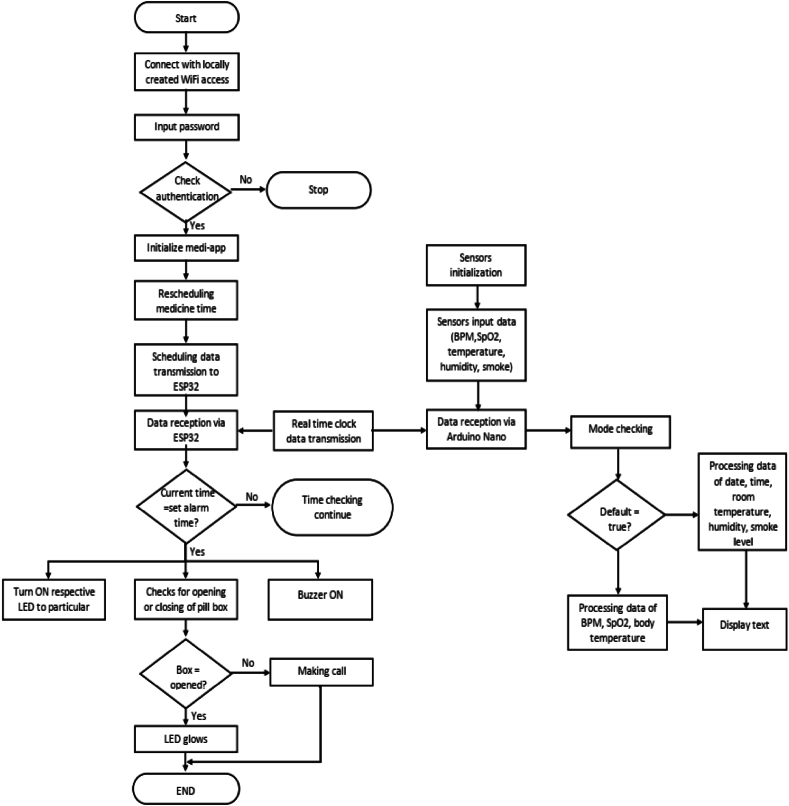
Flow chart of the proposed smart medicine reminder kit.

**Fig. 5:**
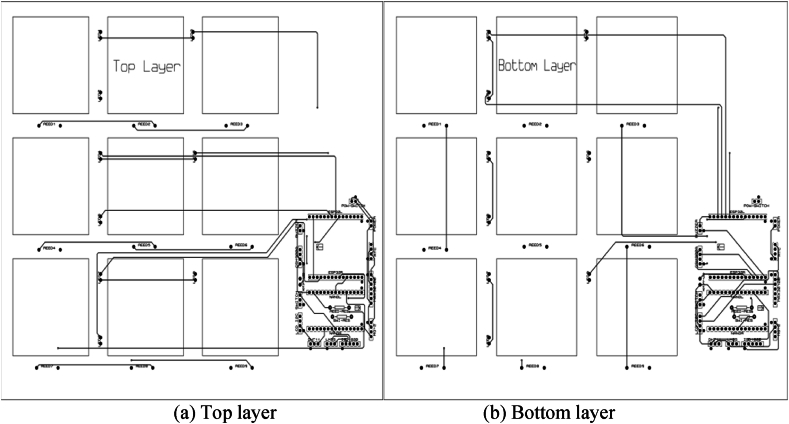
PCB layout of the proposed system.

A temperature sensor, the LM35, is used for sensing human body temperature. The LM35 sensor produces 10 mV changes for every 1 degree of temperature variation. Using Analog pin A0, Arduino receives the output of the temperature sensor and performs the conversion with the help of ADC pins to modify this analog to a digital value of the current temperature. Finally, the temperature is displayed on the LCD display. All the codes for Arduino Nano have been written in the “C" language, and “Arduino IDE” software is used for compiling the sketch. The entire operating process of the proposed smart medicine reminder kit has been depicted in the following flow chart, Fig. 4. The PCB layout of the proposed system has been presented in Fig. 5. Multilayer PCBs with SMD (Surface Mount Device) IC packaging can make the device more compact and energy efficient. We have implemented the proposed system prototype and trialed the system practically for one month. And we have had very good performances in all cases, without any errors. So, the proposed system is also reliable for practical use.

**Fig. 6:**
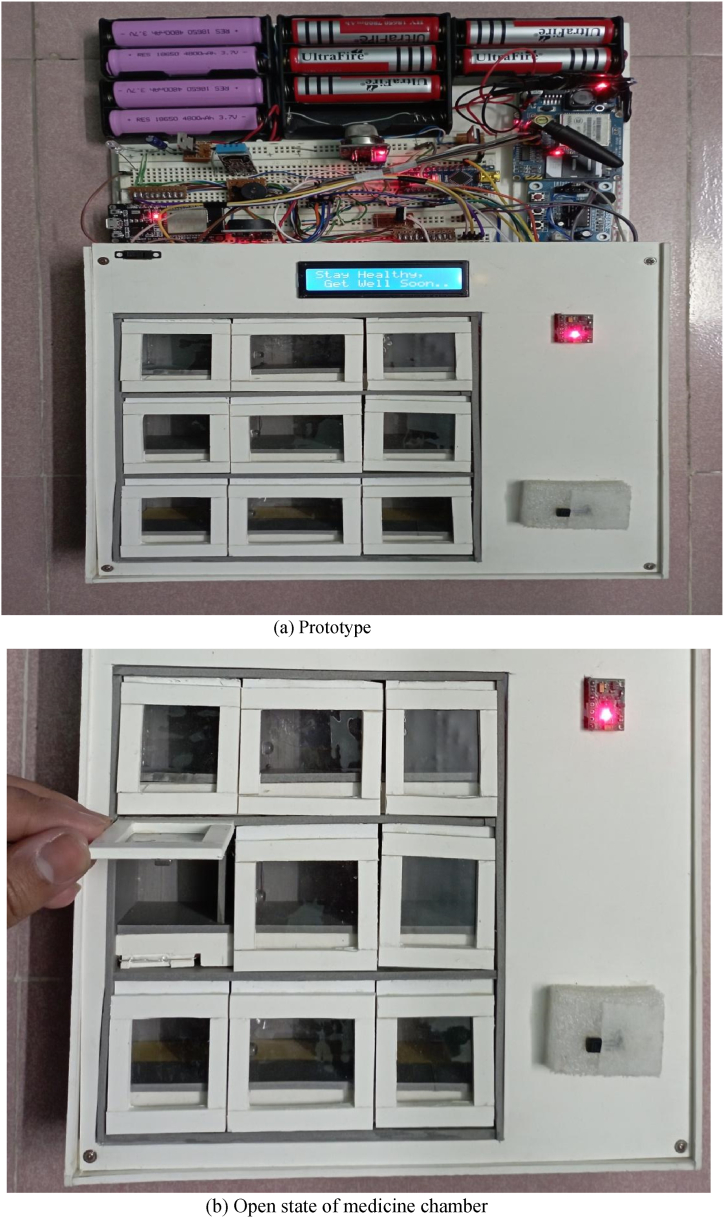
Prototype of the proposed system.

**Fig. 7:**
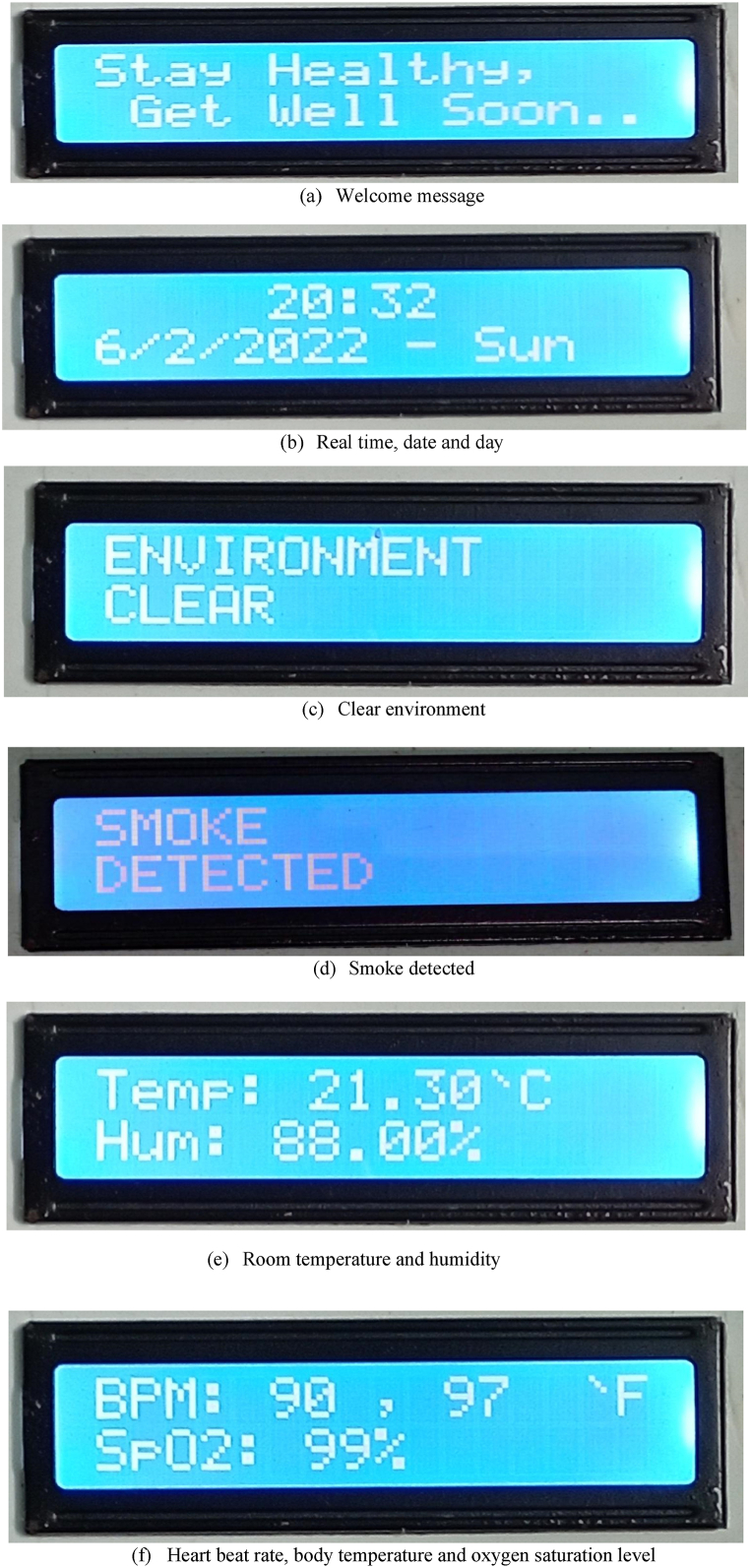

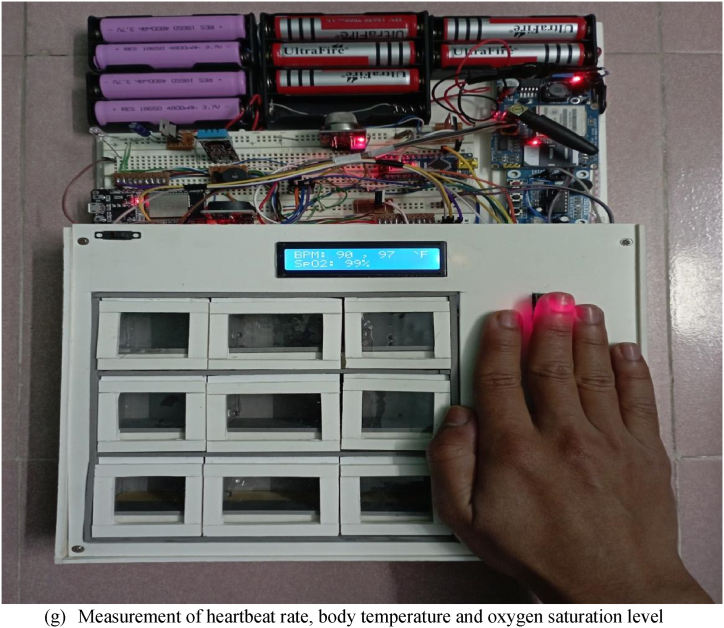
Demonstration of the measurement of all the features of the prototype

The prototype would initially be installed by the manufacturer of the monitoring device or by any family members of the patient. The user's nearest relative or neighbor can then refill the medication box the following time, or the manufacturer may charge a fee for a medication refill service. Always, health and safety are more valuable than money. Additionally, a nation's health ministry or the manufacturer of the proposed system would create a cloud-based national health monitoring server to centrally monitor all such devices in order to guarantee 100% safety and reliability. Our developed prototype of the smart medicine reminder kit with mobile phone calls and its open state of medicine chamber have been presented in Fig. 6(a) and (b), respectively. Fig. 7(a–g) represent the demonstration of measuring all the features of the designed prototype. The implementation cost of a single prototype is about 3225 BDT (approximately 32 USD), as presented in Table 1. If the proposed system is commercially manufactured, then it will be reduced significantly. Therefore, our proposed system is very cost-effective, with some distinct health monitoring features as well as very reliable performance. Without any modification of the existing healthcare system, we can deploy our proposed system to get reminders via mobile phone calls and monitor the aforementioned health monitoring features for senior citizens. A user of the proposed system only needs to install our developed medicine reminder app. Only in order to create a national central health monitoring system is the development of a central server essential, and internet connectivity is needed to make this possible. Another point is that if the government makes a one-time investment to connect all of these devices to form a national health monitoring center, it would ensure the public's health and lower the rate of unintended deaths. The system will only need a small amount of maintenance spending to function.

**Table 1:** Implementation cost of single prototype.

Name of component (quantity)	Price (BDT)
ESP32 (1)	450
Arduino Nano (1)	450
GSM module (1)	800
ISD1820 module (1)	150
MAX30100 Pulse Oximeter and Heart Rate Sensor (1)	250
LM35 body temperature sensor (1)	60
LCD (I2C integrated) (1)	250
DHT11 humidifier + temperature sensor (1)	100
MQ2 gas sensor (1)	100
RTC module (1)	250
IC7805 (2)	15
Buck converter (1)	100
Reed switch (9)	150
LED (10)	15
Buzzer (8)	10
Transistor (2)	5
Capacitor (1)	5
Resistor	10
Switch (1)	5
Cardboard	50
Total	3225 BDT (Approx. 32 USD)

At this stage, every patient needs to use a different piece of the prototype of the developed system. This is a limitation of our system. There is no opportunity to use a single prototype by two or more patients at a time. In the future, we would try to modify and develop a system where a single prototype could be used by multiple users at a time. It will also reduce the cost for a family if there is more than one patient in a single family. In that case, a single prototype could serve all the multiple family members at a time.

## 4 Conclusion

Statistics show that one of the most difficult problems that has to be solved is getting an elderly person to take the proper medication at the right time. In this study, a mobile app-based portable intelligent medication reminder system for home use has been created. The low-cost prototype is equipped with the ESP32, RTC, LCD screen, ATmega328P, Arduino Nano, GSM, MAX30100, LM35, etc. If the patient does not open the door of the prescribed prescription box after the alarm, the smart system can call the patient at a specified number after 5 min. The user finds the suggested system appealing because of this feature. In addition to serving as a reminder, it also looks after the elderly through routine health checks. This system is appropriate for older, independently living people or households where all the family members are busy with their own work. After developing the system, it is tested by inputting a medication schedule using the developed app. The implemented prototype shows very good performance for all the possible cases. The system is designed with nine chambers, and the number of chambers can be increased as per requirement. Further, there is an opportunity to upgrade the system. The developed system provides some information about our health locally, i.e., on the LCD display. We have a plan to upgrade the gadget in the future to include a multi-user cloud-based monitoring system, enabling telemedicine applications. Furthermore, as the GSM system is one of the system's essential components, it can be incorporated as a further feature to make real-time phone calls with someone else, perhaps reducing the perceived distance between the patient and the doctor.

## Funding statement

The research was supported by the University Grants Commission of Bangladesh, FY: 2023–2024.

## Data availability statement

Data included in article/supp. material/referenced in article.

## CRediT authorship contribution statement

**Liton Chandra Paul:** Writing – review & editing, Writing – original draft, Visualization, Validation, Supervision, Software, Resources, Project administration, Methodology, Investigation, Funding acquisition, Formal analysis, Data curation, Conceptualization. **Sayed Shifat Ahmed:** Writing – original draft, Visualization, Validation, Software, Methodology, Investigation, Formal analysis, Data curation, Conceptualization. **Tithi Rani:** Writing – review & editing, Writing – original draft, Visualization, Validation, Supervision, Software, Resources, Project administration, Methodology, Investigation, Formal analysis, Data curation, Conceptualization. **Md Ashraful Haque:** Writing – review & editing, Validation, Software, Methodology, Investigation, Formal analysis. **Tushar Kanti Roy:** Writing – review & editing, Validation, Supervision, Resources, Project administration, Methodology, Formal analysis, Data curation. **Md Najmul Hossain:** Writing – review & editing, Validation, Software, Methodology, Formal analysis, Data curation. **Md Azad Hossain:** Writing – review & editing, Visualization, Validation, Resources, Formal analysis, Conceptualization.

## Declaration of competing interest

The authors declare that they have no known competing financial interests or personal relationships that could have appeared to influence the work reported in this paper.
